# Comparative analysis of del Nido cardioplegia versus blood cardioplegia in isolate coronary artery bypass grafting

**DOI:** 10.1186/s13019-024-02853-1

**Published:** 2024-07-13

**Authors:** Soojin Lee, Joon Chul Jung, Hyoung Woo Chang, Jae Hang Lee, Dong Jung Kim, Jun Sung Kim, Cheong Lim

**Affiliations:** 1 Department of Thoracic and Cardiovascular Surgery, School of Medicine, Pusan National University, Biomedical Research Institute, Pusan National University Hospital, Busan, Republic of Korea; 2 Department of Thoracic and Cardiovascular Surgery, Seoul National University Bundang Hospital, Seoul National University College of Medicine, Bundang-gu, Seongnam-si, 13620 Gyeonggi-do Republic of Korea

**Keywords:** Cardioplegia, Coronary artery bypass grafting, Cardiopulmonary bypass

## Abstract

**Background:**

This study examined the efficacy of del Nido cardioplegia compared with traditional blood cardioplegia in adult cardiac surgery for isolated coronary artery bypass grafting by evaluating the early postoperative outcomes.

**Methods:**

A total of 119 patients who underwent isolated conventional coronary artery bypass grafting were enrolled and divided into two groups (del Nido cardioplegia group [*n* = 36] and blood cardioplegia group [*n* = 50]) based on the type of cardioplegia used. This study compared the preoperative characteristics, intraoperative data, and early postoperative outcomes. Further subgroup analyses were conducted for high-risk patient groups.

**Results:**

The 30-day mortality and morbidity rates were not significantly different between groups. The del Nido cardioplegia group exhibited advantageous myocardial protection outcomes, demonstrated by a significantly smaller rise in Troponin I levels post-surgery (2.8 [-0.4; 4.2] vs. 4.5 [2.9; 7.4] ng/mL, *p* = 0.004) and fewer defibrillation attempts during weaning off of cardiopulmonary bypass (0.0 ± 0.2 vs. 0.4 ± 1.1 times, *p* = 0.011) when compared to the blood cardioplegia group. Additionally, the del Nido group achieved a reduction in surgery duration, as evidenced by the reduced aortic cross-clamping time (64.0 [55.5; 75.5] vs. 77.5 [65.0; 91.0] min, *p* = 0.001) and total operative time (287.5 [270.0; 305.0] vs. 315.0 [285.0; 365.0] min, *p* = 0.008). Subgroup analyses consistently demonstrated that the del Nido cardioplegia group had a significantly smaller postoperative increase in Troponin I levels across all subgroups (*p* < 0.05).

**Conclusions:**

del Nido cardioplegia provided myocardial protection and favorable early postoperative outcomes compared to blood cardioplegia, making it a viable option for conventional coronary artery bypass grafting. Establishing a consensus on the protocol for Del Nido cardioplegia administration in adult surgeries is needed.

## Background

The choice of cardioplegic solution in cardiac surgery depends on various factors such as the surgery type and the surgeon’s preference and expertise. While blood cardioplegia has been the traditional choice and remains widely used, del Nido cardioplegia is gaining popularity in adult cardiac surgeries following its proven safety in pediatric cardiac procedures [1, 2]. Echoing global trends, our centre has been using del Nido cardioplegia for coronary artery bypass grafting (CABG) since 2021, transitioning from the previously used blood cardioplegia. However, not all surgeons have used this technique. Similarly, the optimal cardioplegic solution is still under research, as the field has not yet reached a consensus [3].

Myocardial protection is a crucial consideration in various heart surgeries, particularly CABG for ischaemic heart disease. Studies on the safety and efficacy of del Nido cardioplegia for CABG are limited. Existing studies often combine results from multiple surgeons, necessitating careful interpretation of the different surgical strategies used by the included surgeons [4, 5].

In this study, we aimed to compare the early postoperative results between del Nido and traditional blood cardioplegia in CABG performed by a single surgeon at our institution, offering a unique perspective on the efficacy of these two cardioplegic solutions in a controlled setting. Our hypothesis was that del Nido cardioplegia would demonstrate equivalent early postoperative outcomes to those of traditional blood cardioplegia while also offering a more feasible cardioprotective strategy.

## Methods

### Ethical statement

This observational clinical study did not require clinical trial registration. The research was conducted by reviewing medical records and the analyzed data was de-identified to protect the anonymity and non-identifiability of the patients. This study was approved by the Institutional Review Board of the Seoul National University Bundang Hospital (IRB No. B-2307-838-105). The requirement for informed consent was waived because the analysis was conducted retrospectively, using electronic medical records.

### Patients and data collection

**Fig. 1:**
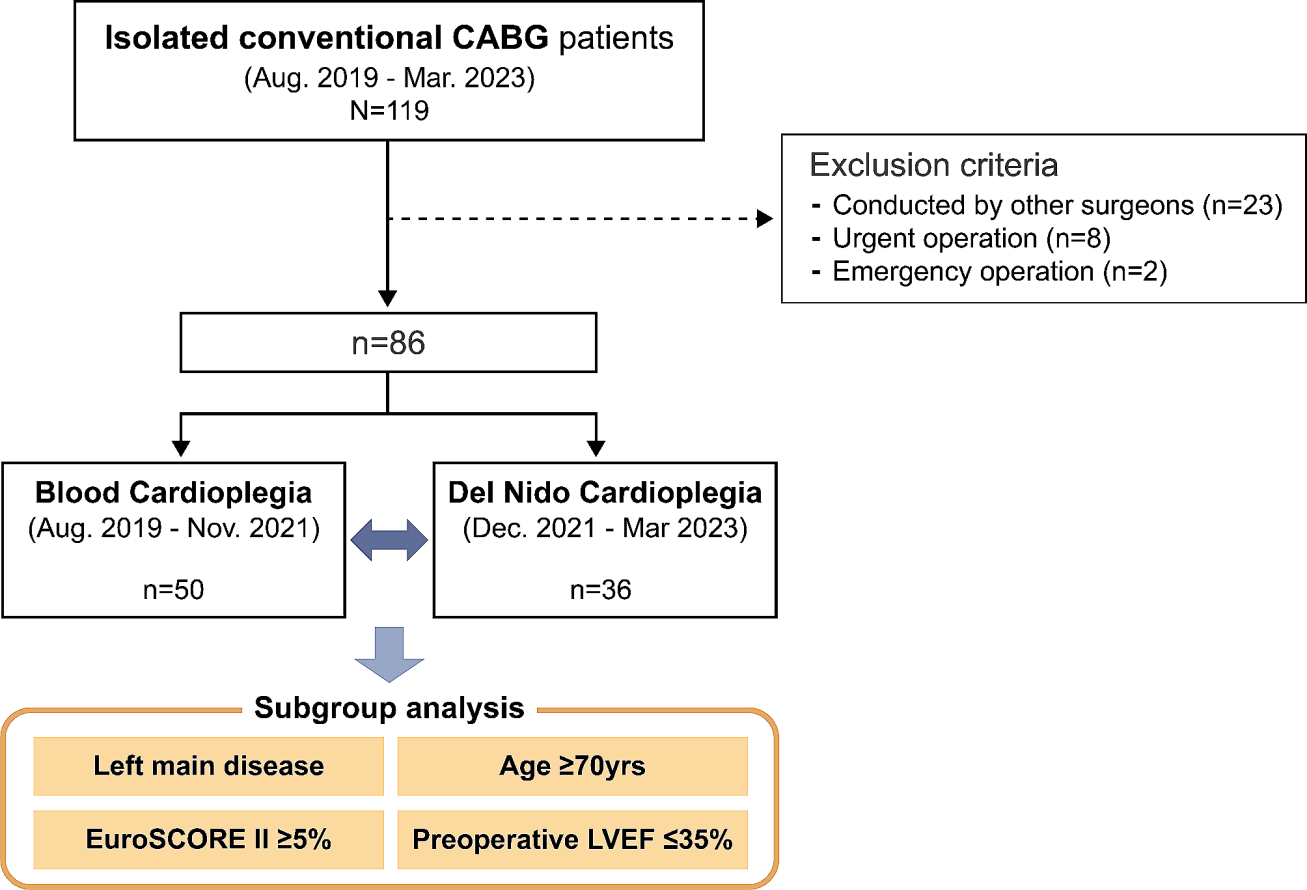
Study flowchart CABG, coronary artery bypass grafting; EuroSCORE, European System for Cardiac Operative Risk Evaluation; LVEF, left ventricular ejection fraction

This retrospective study analysed the electronic medical records of patients who underwent isolated conventional CABG at our institution between August 2019 and March 2023. The focus was on the key protocol changes implemented by a single cardiac surgeon in December 2021, transitioning from traditional blood cardioplegia to del Nido cardioplegia for CABG. To ensure a consistent and homogeneous dataset reflective of the surgeon’s practice, we excluded cases performed by other surgeons, amounting to 23 exclusions. The cardiac surgeon conducting the operations has accrued over ten years of experience in independently performing CABG, starting before the onset of the study period. Furthermore, the study was narrowed to elective surgeries by omitting urgent (*n* = 8) and emergent (*n* = 2) operations. In our center, urgent and emergent CABG is defined as surgery conducted within two days and 12 h after the recognition of the urgency of the disease, respectively. This selective criterion resulted in a cohort of 86 patients (mean age: 67.0 ± 11.0 years, 81.0% male) for detailed analysis and comparison of the two cardioplegia techniques in elective CABG procedures (Fig. 1).

Data collection for this study was comprehensive and covered patient characteristics, intraoperative details, and early postoperative outcomes. We meticulously recorded patient demographics and clinical profiles, including sex, age, underlying diseases, history of previous cardiac surgery, and European System for Cardiac Operative Risk Evaluation (EuroSCORE) II. Additional preoperative data on the type of ischaemic heart disease, preoperative echocardiographic findings, and status of extracorporeal membrane oxygenation or intra-aortic balloon pump use were also collected.

The intraoperative data included the type and quantity of cardioplegic solutions used, estimated blood loss, intraoperative transfusions, total operative time, duration of cardiopulmonary bypass (CPB) support and aortic cross-clamping (ACC), and the need for defibrillation or pacing during CPB weaning. Details of the grafts used for CABG, including the number of distal anastomoses and the use of Y-composite grafts, were also collected.

Early postoperative outcomes included complications during hospital stay, need for postoperative extracorporeal membrane oxygenation or intra-aortic balloon pump support, total duration of hospital and intensive care unit (ICU) stay, postoperative ventilator-dependent duration, incidence of reintubation, 30-day mortality, 1-year Major Adverse Cardiac and Cerebrovascular Events and readmission rates. Additionally, to assess myocardial protection, we evaluated peak cardiac enzyme levels (CK-MB and troponin I) and postoperative echocardiographic findings.

### Patient selection criteria of conventional CABG and myocardial protection strategy

In our centre, patients are selected for conventional CABG based on a preoperative low left ventricular ejection fraction (LVEF) and/or a poor distal coronary bed. Therefore, approximately 20% of patients with ischaemic heart disease who require CABG undergo conventional CABG.

All patients underwent conventional CABG under general anaesthesia. The procedure began with a routine median sternotomy, followed by central cannulation, which was performed after harvesting the grafts. For patients in the blood cardioplegia group, the initial administration consisted of 1,000 mL of blood cardioplegia at a 4:1 blood dilution, delivered in an antegrade fashion at 4 °C. This was supplemented with 200 mL doses delivered in a retrograde fashion every 15–20 min as required. At the conclusion of the final anastomosis, 500 mL of warm blood was infused in a retrograde dose. In contrast, patients in the del Nido cardioplegia group received a single antegrade 1,000 mL del Nido dose at a 1:4 blood dilution and 4 °C [1]. If the ACC time was anticipated to exceed 90 min, a supplementary 500 mL dose was administered in a retrograde fashion 60 min after the initial dose. Both groups were subjected to mild hypothermia (32–34 °C) and topical cooling methods during the procedure.

### Comparison between blood cardioplegia versus Del Nido Cardioplegia

In this study, the participants were categorised into two cohorts based on the type of cardioplegic solution used during surgery. The blood cardioplegia group consisted of patients who underwent surgery using blood cardioplegia between August 2019 and November 2021. The second group, the del Nido cardioplegia group, included patients treated with del Nido cardioplegia between December 2021 and March 2023. This division was based on the transition between the two cardioplegic techniques. A comprehensive comparative analysis was performed between the two groups. This analysis focused on evaluating the preoperative patient characteristics, intraoperative variables, and early postoperative outcomes.

Please refer to Table 1 for the compositions of the blood cardioplegia and del Nido cardioplegia used in this study.

**Table 1:** Composition of blood cardioplegia and del Nido cardioplegia

	Blood cardioplegia	Del Nido cardioplegia
**Base solution (1 L)**	Plegisol	Plasma-lyte A
**Blood : Crystalloid ratio**	4:1	1:4
**Potassium**	80 mEq	26 mEq
**Bicarbonate**	30 mEq	13 mEq
**Mannitol 20%**	12.5 g	3.3 g
**Lidocaine 1%**	-	0.13 g
**Magnesium**	-	2 g

### Statistical analysis

The baseline characteristics of the two groups were compared and differences in intraoperative data and postoperative outcomes were assessed. For continuous variables, comparisons were made using either the independent t-test or the Wilcoxon rank-sum test. Categorical variables were analysed using the chi-squared test or Fisher’s exact test. Subgroup analyses mirrored the overall cohort’s analytical approach and were conducted on four distinct high-risk groups to evaluate the efficacy of del Nido cardioplegia compared to blood cardioplegia, defined by specific preoperative criteria: the presence of left main disease, age of ≥ 70 years, LVEF of ≤ 35%, and a EuroSCORE II ≥ 5%. Statistical significance was set at a p-value of less than 0.05. All statistical analyses were performed using R version 4.2.2 (R Core Team, 2020, available at http://cran.r-project.org).

## Results

### Baseline patient characteristics

Table 2 shows the baseline patient characteristics. The study included 50 and 36 patients in the blood and del Nido cardioplegia groups, respectively. The median EuroSCORE II was 2.0% (with a range of 0.9–3.7%) for blood cardioplegia recipients and 1.9% (ranging from 1.2 to 3.2%) for those in the del Nido cardioplegia group. There were no notable differences in the baseline characteristics between the groups.

**Table 2:** Preoperative characteristics

Variables		BC (*n* = 50)	DC (*n* = 36)	*P* value
**Age**		64.4 [59.5;75.0]	67.6 [63.1;75.7]	0.398
**Male, years**		42 (84.0%)	27 (75.0%)	0.448
**BMI, kg/m** ^**2**^		23.2 [21.6;24.8]	24.9 [21.6;26.3]	0.284
**EuroSCORE II, %**		2.0 [0.9;3.7]	1.9 [1.2;3.2]	0.611
**Underlying disease**				
	**Hypertension**	36 (72%)	26 (72.2%)	> 0.999
	**Diabetes**	35 (70%)	28 (77.8%)	0.578
	**CVA**	9 (18%)	7 (19.4%)	> 0.999
	**CKD**	12 (24%)	9 (25%)	> 0.999
	**COPD**	2 (4%)	3 (8.3%)	0.704
**Type**				
	**Silent MI**	4 (8%)	1 (2.8%)	0.580
	**Stable angina**	6 (12%)	5 (13.9%)	> 0.999
	**Unstable angina**	17 (34%)	12 (33.3%)	> 0.999
	**NSTEMI**	22 (44%)	15 (41.7%)	> 0.999
	**STEMI**	1 (2%)	3 (8.3%)	0.391
**Preoperative ECMO**		0	1 (2.8%)	0.868
**Reoperation**		0	1 (2.8%)	0.868
**Echocardiography**				
	**LVEF, %**	44.1 [28.1;57.9]	40.5 [30.4;55.3]	0.733
	**RWMA**	39 (79.4%)	27 (75%)	0.811

BC, blood cardioplegia; DC, del Nido cardioplegia; BMI, Body mass index; EuroSCORE, European System for Cardiac Operative Risk Evaluation; CVA, cerebrovascular accident; CKD, chronic kidney disease; COPD, chronic obstructive pulmonary disease; STEMI, ST-elevation myocardial infarction; NSTEMI, non-ST-elevation myocardial infarction; MI, myocardial infarction; ECMO, extracorporeal membrane oxygenation; LVEF, left ventricular ejection fraction; RWMA, regional wall motion abnormality

Values are presented as means ± standard deviation, median [range], or number (%)

### Intraoperative data

Table 3 details the intraoperative outcomes for both patient groups. In the blood cardioplegia group, cardioplegia was administered in both an antegrade and retrograde manner multiple times (mean 6.1 ± 1.4 times), whereas most of the patients in the del Nido cardioplegia group received a single antegrade administration, resulting in significantly less total cardioplegia doses. The volume of cardioplegia used was greater in the blood cardioplegia group for both antegrade (1160 ± 220 mL) and retrograde (1839 ± 535 mL) administrations compared to the del Nido cardioplegia group (antegrade: 997 ± 191 mL, retrograde: 626 ± 269 mL) (*p* < 0.001). The ACC and operative time were notably shorter in the del Nido group. Furthermore, in the del Nido group, the number of defibrillations during CPB weaning was significantly reduced.

**Table 3:** Intraoperative data

Variables		BC (*n* = 50)	DC (*n* = 36)	*P* value
**Cardioplegia volume, mL**				
	**Antegrade**	1160 ± 220	997 ± 191	< 0.001
	**Retrograde**	1839.5 ± 535.3	626.4 ± 269.0	< 0.001
**Cardioplegia, number of doses**				
	**Antegrade**	1.0 ± 0.0	1.0 ± 0.2	0.324
	**Retrograde**	6.1 ± 1.4	1.1 ± 0.4	0.000
**CPB time, min**		116.0 [100.0;137.0]	107.5 [91.5;125.5]	0.135
**Aortic clamp time, min**		77.5 [65.0;91.0]	64.0 [55.5;75.5]	0.001
**Operative time, min**		315.0 [285.0;365.0]	287.5 [270.0;305.0]	0.008
**Number of defibrillation, times**		0.4 ± 1.1	0.0 ± 0.2	0.011
**Pacing needed after CPB weaning**		7 (14.0%)	5 (13.9%)	1.000
**Number of distal anastomosis**		4.3 ± 0.7	4.5 ± 0.8	0.230
**Y-composite graft**		45 (90.0%)	27 (75.0%)	0.118
**Transfusion amount**				
	**pRBC**	4.0 [3.0; 5.0]	4.0 [2.0; 6.0]	0.750
	**FFP**	3.0 [2.0; 3.0]	2.0 [1.0; 3.0]	0.198
	**PC**	0.0 [0.0; 0.0]	0.0 [0.0; 0.0]	0.396

BC, blood cardioplegia; DC, del Nido cardioplegia; CPB, cardiopulmonary bypass; pRBC, packed red blood cells; FFP, fresh frozen plasma; PC, platelet concentrate

Values are presented as means ± standard deviation, median [range], or number (%)

### Postoperative outcomes and myocardial protection

Table 4 shows the comparison of postoperative outcomes between the two groups. There were no statistically significant differences in the 30-day mortality or postoperative complications between the groups.

**Table 4:** Postoperative outcomes

Variables		BC (*n* = 50)	DC (*n* = 36)	*P* value
**Mortality, < 30 days**		3 (6.0%)	2 (5.6%)	> 0.999
**ICU stay, days**		1.8 [0.9; 3.9]	2.0 [1.0; 3.1]	0.578
**Hospital stay, days**		9.0 [7.0;14.0]	9.0 [7.0;15.0]	0.866
**New onset atrial fibrillation**		3 (6.0%)	2 (5.7%)	> 0.999
**MACCE, < 1 year**	**Total**	11 (22.0%)	6 (16.7%)	0.735
	Stroke	3	2	
	Myocardial infarction	1	1	
	Death	7	3	
**LCOS**		1 (2.0%)	1 (2.8%)	> 0.999
**Postoperative ECMO**		4 (8.0%)	3 (8.3%)	> 0.999
**CK-MB, ng/mL**				
	**Peak value**	26.4 [19.1;38.4]	25.5 [17.8;30.9]	0.286
	**Difference (peak value – preoperative value)**	18.8 [10.3;31.7]	14.4 [5.8;26.7]	0.068
**Troponin I, ng/mL**				
	**Peak value**	5.0 [3.5; 9.0]	6.7 [3.8;10.3]	0.300
	**Difference (peak value - preoperative value)**	4.5 [2.9; 7.4]	2.8 [-0.4; 4.2]	0.004
**LVEF, %**		43.8 ± 14.6	46.3 ± 12.9	0.420
**LVEF difference (peak value- preoperative value), %**		0.2 ± 8.0	3.6 ± 8.8	0.067

BC, blood cardioplegia; DC, del Nido cardioplegia; ICU, Intensive care unit; MACCE, major adverse cardiac and cerebrovascular events; LCOS, low cardiac output syndrome; ECMO, extracorporeal membrane oxygenation; LVEF, left ventricular ejection fraction

Values are presented as means ± standard deviation, median [range], or number (%)

The change in Troponin I levels, calculated as the difference between the postoperative peak and preoperative levels, was significantly lower in the del Nido cardioplegia group than in the blood cardioplegia group (*p* = 0.004). While the initial postoperative LVEF measurements obtained during the first echocardiography after surgery and the improvement in LVEF (derived from the difference between the first postoperative LVEF and the preoperative LVEF) were higher in the del Nido cardioplegia group, these differences were not statistically significant.

### Subgroup analyses

To verify whether the efficacy of del Nido cardioplegia was consistent even in high-risk groups, a comparison of outcomes between the del Nido and blood cardioplegia groups was conducted in four subgroups, as described in the Methods section (Table 5). While early mortality and morbidity rates were comparable between the two cardioplegic solutions across high-risk subgroups, del Nido cardioplegia exhibited a more favourable influence on Troponin I changes in all subgroups. Additionally, it showed significantly better improvement in postoperative LVEF in patients with left main disease and less requirement for defibrillation in patients aged ≥ 70 years.

## Discussion

Our findings revealed that both groups had equivalent 30-day mortality and morbidity rates. Similarly, the incidence of new-onset atrial fibrillation, as well as the duration of hospital and ICU stays, showed no significant differences between the groups. These results are consistent with a previous study that found similar early postoperative outcomes and lengths of hospital and ICU stay among propensity-matched patients undergoing isolated CABG, comparing those who received del Nido cardioplegia to those who received blood cardioplegia [6].

The importance of strategies for myocardial protection in cardiac surgeries involving CPB has been highlighted because of the risk of acute ischaemic damage to the ACC and subsequent myocardial reperfusion injury upon release of the clamp. Hence, the choice and composition of cardioplegic solutions are areas of significant interest, with blood cardioplegia emerging as the preferred option worldwide [2]. Initially adopted in paediatric surgeries, del Nido cardioplegia has been recognised for its safety and efficacy, leading to its increasing application in adult cardiac procedures [1]. However, debate over the most effective cardioplegia treatment for adults persists [3]. This study aimed to assess the effectiveness of del Nido cardioplegia against traditionally used blood cardioplegia in patients undergoing CABG, examining not only early postoperative clinical outcomes but also indicators of myocardial protection. Diverging from prior studies that primarily evaluated postoperative LVEF, this study broadened its analysis to include postoperative LVEF, LVEF change (comparing pre- and post-surgery levels), cardiac enzyme levels before and after the operation, and the number of defibrillations needed during CPB weaning [6, 7].

Rat studies have validated the cardioprotective mechanism of del Nido cardioplegia, particularly its efficacy in averting calcium-induced hypercontraction and reperfusion injuries due to its key components, including magnesium and lidocaine [8]. Additionally, molecular research has demonstrated the role of lidocaine in myocardial protection through the upregulation of genes associated with anti-apoptosis, diminished intracellular calcium binding, reduced inflammation, improved oxygen delivery, and enhanced cell survival [9].

In our study, del Nido cardioplegia demonstrated potential superiority in myocardial protection, as evidenced by the detailed assessment of both functional and biochemical markers. While postoperative LVEF changes, serving as a functional indicator of myocardial protection, showed comparable results between the two groups, the del Nido group outperformed the blood cardioplegia group in terms of troponin I level changes, a key biochemical marker of myocardial protection (2.8 [-0.4; 4.2] vs. 4.5 [2.9; 7.4] ng/mL, *p* = 0.004). Another noteworthy finding from our study is that the group receiving del Nido cardioplegia required defibrillation less frequently during the weaning process from CPB, hinting at enhanced myocardial protection throughout the reperfusion period compared to the blood cardioplegia group. Arrhythmias necessitating interventions, such as defibrillation during CPB weaning, may arise from multiple sources, including insufficient myocardial protection [10, 11].

Our research further supports the idea that del Nido cardioplegia enhances the efficiency of CABG surgeries compared to traditional blood cardioplegia. The group that received del Nido cardioplegia required significantly lower volumes of cardioplegic solution for both antegrade and retrograde deliveries. Using greater amounts of cardioplegia requires extended interruptions during the coronary anastomosis, which lengthens the duration of the procedure. Furthermore, del Nido cardioplegia requires significantly lower maintenance doses, which results in fewer disruptions to the surgical process. Additionally, the del Nido cardioplegia group required fewer defibrillations, which likely reduced surgical interruptions. Consequently, these relatively fewer interruptions contributed to a decrease of 13.5 min in the ACC time and approximately 28 min in the total surgical duration. Given that extended operation times are associated with increased hospital costs, the use of del Nido cardioplegia may not only streamline the surgical process, but also mitigate the financial impact on patients, aligning with the growing focus on reducing healthcare expenses [12, 13].

Conventional approaches for treating multivessel coronary artery disease often employ a combination of antegrade and retrograde cardioplegic delivery. However, we derived comparable postoperative outcomes with only the antegrade infusion method for cardioplegia compared to inducing cardiac arrest with antegrade infusion and maintaining it with retrograde infusion, which is in agreement with previous research findings [14]. However, existing studies have explored the impact of cardioplegic delivery methods on postoperative outcomes, yielding results that diverge from ours. A clinical trial that assessed postoperative myocardial function in patients with three-vessel disease undergoing CABG found that those who received retrograde cardioplegia showed better preservation of the left ventricular stroke work index than those who underwent antegrade delivery [15]. Additionally, retrospective analyses examining early clinical outcomes in three-vessel disease patients, comparing antegrade-only to combined antegrade and retrograde cardioplegia delivery, indicated a superior myocardial protective effect in the latter group [16, 17]. Notably, these studies utilised cardioplegia solutions without lidocaine, such as blood cardioplegia or St. Thomas’ Hospital solution, unlike the lidocaine-containing del Nido cardioplegic solution, known for its myocardial protection benefits [8, 9, 18]. Thus, it may be inferred that the type of cardioplegic solution used for multivessel disease during CABG, rather than the route of administration, plays a pivotal role in myocardial protection. Investigating the optimal delivery method for del Nido cardioplegia in future studies could provide a definitive answer to this debate.

Additional analyses of high-risk surgical patients showed results consistent with overall patient outcomes. There were no significant differences in the early postoperative mortality and morbidity between the del Nido and blood cardioplegia groups across all subgroups. However, the del Nido cardioplegia group exhibited lower Troponin I increment, particularly in patients with left main disease. This subgroup showed a significantly greater improvement in postoperative LVEF in the del Nido group. For those aged ≥ 70 years, the requirement for defibrillation was lower in the del Nido cardioplegia group than in the blood cardioplegia group.

The limitations of this study are its retrospective design and the limited number of participants, which was a consequence of our stringent selection criteria for conventional CABG in patients with ischaemic heart disease, as detailed in the Methods section. Despite these constraints, this study represents an inaugural attempt to evaluate the effectiveness of del Nido cardioplegia in conventional CABG through a series performed by a single surgeon, potentially minimising the variability and bias that often accompany the data from previous studies involving multiple surgeons. Nonetheless, to achieve broader and more universally applicable insights, future research will require large-scale prospective studies. In addition, caution is advised when concluding the superiority of del Nido cardioplegia in high-risk patients because of the small sample size in the subgroup analysis.

## Conclusion

Del Nido cardioplegia, when compared with blood cardioplegia in conventional CABG, showed similar early postoperative outcomes, notable myocardial protection, and a shorter surgical time by maintaining a steady flow of operation.

## Data Availability

No datasets were generated or analysed during the current study.

## Abbreviations

CABG: Coronary artery bypass grafting
LVEF: Left ventricular ejection fraction
EuroSCORE: European System for Cardiac Operative Risk Evaluation
CPB: Cardiopulmonary bypass
ACC: Aortic cross-clamping
ICU: Intensive care unit
